# Beyond Gastric Specificity: V-Set and Immunoglobulin Domain-Containing 1 (VSIG1) in Digestive Tract Tumors

**DOI:** 10.3390/cancers18050867

**Published:** 2026-03-08

**Authors:** Catalin-Bogdan Satala, Gabriela Patrichi, Alina-Mihaela Gurau, Andreea Onofrei (Popa), Daniela Mihalache

**Affiliations:** 1 “Dunărea de Jos” University of Galati, Faculty of Medicine and Pharmacy, Medical and Pharmaceutical Research Center, 800008 Galati, Romania; catalin.satala@ugal.ro (C.-B.S.); andreea.onofrei@ugal.ro (A.O.); daniela.mihalache@ugal.ro (D.M.); 2 Department of Pathology, Clinical County Emergency Hospital Braila, 810325 Braila, Romania; 3 The Doctoral School of Medicine and Pharmacy, “George Emil Palade” University of Medicine, Pharmacy, Science and Technology, 540142 Targu Mures, Romania; 4 The School for Doctoral Studies in Biomedical Sciences, “Dunarea de Jos” University of Galati, 800008 Galati, Romania; alina.gurau@ugal.ro

**Keywords:** VSIG1, V-set and immunoglobulin domain-containing 1, epithelial differentiation, gastric cancer, digestive tract neoplasms, immunoglobulin superfamily, phenotypic plasticity

## Abstract

V-set and immunoglobulin domain-containing 1 (VSIG1) is a member of the immunoglobulin superfamily that has attracted increasing attention as a differentiation-associated protein in gastrointestinal neoplasia. Although initially described as a gastric-specific marker, accumulating evidence indicates that VSIG1 more accurately reflects gastric-enriched epithelial differentiation rather than strict anatomical origin. This conceptual shift has implications for phenotype-oriented tumor classification and diagnostic interpretation in the context of lineage plasticity. A structured and transparently reported literature search was conducted in PubMed/MEDLINE, Web of Science, and Scopus, covering studies published between 2000 and 2024. Eligible studies included original research and relevant reviews evaluating VSIG1 expression in normal tissues and digestive tract tumors, with emphasis on immunohistochemical patterns and clinicopathological correlations. In gastric cancer, VSIG1 expression consistently correlates with preserved glandular architecture and epithelial differentiation, whereas reduced or absent expression accompanies dedifferentiation and architectural disorganization. Outside the stomach, VSIG1 positivity is uncommon but reproducible in tumors exhibiting gastric-type or mixed differentiation, including settings of hepato-gastric phenotypic overlap. These patterns support interpretation of VSIG1 as a context-dependent indicator of lineage engagement and differentiation state rather than tumor origin or aggressiveness. Current data on independent prognostic value are limited and partially conflicting, and predictive roles remain unsupported, while functional data remain limited.

## Simple Summary

VSIG1 is a protein that is normally expressed at higher levels in the stomach mucosa and is involved in maintaining epithelial differentiation. In recent years, VSIG1 has been increasingly studied in cancer, particularly in tumors of the gastrointestinal tract. While it was initially considered a stomach-specific marker, accumulating evidence shows that VSIG1 can also be expressed in other epithelial tumors, especially those showing gastric-type features. In this review, we summarize current knowledge on VSIG1 expression, with a focus on gastric and non-gastric digestive tract tumors. We discuss how VSIG1 expression relates to epithelial differentiation and tumor plasticity rather than tumor origin or aggressiveness. Understanding the biological context of VSIG1 expression may help pathologists and researchers interpret tumor phenotypes more accurately and avoid overestimating the specificity or clinical significance of this marker.

## 1. Introduction

Despite major advances in molecular oncology, the identification and clinical implementation of reliable biomarkers in digestive tract cancers remain challenging. Many proposed biomarkers demonstrate limited specificity, context dependency, or inconsistent reproducibility across tumor types and patient cohorts. In this landscape, increasing attention has shifted from broadly expressed molecular alterations toward lineage- and differentiation-associated markers, which reflect the biological identity of epithelial cells and their deviations during tumorigenesis [1].

Epithelial differentiation represents a fundamental organizing principle of the gastrointestinal tract [2]. Each segment of the digestive system is characterized by highly specialized epithelial programs that govern tissue architecture, cellular polarity, and functional output. During carcinogenesis, these differentiation programs are frequently disrupted, resulting in phenotypic plasticity, loss of tissue-specific identity, and acquisition of aggressive tumor features [3]. Importantly, such alterations do not occur uniformly but follow organ-specific and lineage-dependent trajectories, emphasizing the need for biomarkers that capture this biological context [4]. In this context, tissue-restricted differentiation markers offer unique insights. Rather than acting as universal indicators of malignancy, these molecules provide information about cellular origin, differentiation status, and phenotypic stability [5]. Their expression or loss of it can therefore illuminate key aspects of tumor biology that extend beyond simple diagnostic classification, including tumor progression, plasticity, and evolutionary potential [6]. V-set and immunoglobulin domain-containing 1 (VSIG1) has emerged as a particularly intriguing example of such a marker [7]. Functional studies in experimental models have suggested a role for VSIG1 in maintaining gastric epithelial differentiation, while immunohistochemical studies demonstrate reduced or lost expression in poorly differentiated gastric tumors [7,8]. Unlike conventional oncogenic drivers or proliferation markers, VSIG1 appears to be closely linked to the maintenance of epithelial identity, positioning it at the intersection between differentiation biology and tumor pathogenesis [7]. Although most studies to date have focused on the stomach, accumulating evidence suggests that VSIG1 expression may extend beyond its organ of origin under pathological conditions [9]. Reports of VSIG1 expression in non-gastric digestive tract neoplasms—particularly in tumors exhibiting aberrant or ectopic gastric differentiation—raise important questions regarding its biological significance and diagnostic interpretation [10].

These observations challenge a strictly organ-centric view of VSIG1 and instead point toward a broader role in lineage commitment and epithelial identity within digestive tract neoplasia. Reframing VSIG1 as a marker of differentiation rather than of strict anatomical origin has important conceptual and practical implications. Misclassification of differentiation-associated proteins as tissue-specific markers may lead to diagnostic overinterpretation, particularly in tumors with mixed or aberrant phenotypes. In the era of lineage plasticity and phenotype-driven tumor classification, biomarkers must be interpreted within dynamic differentiation programs rather than fixed organ boundaries. Recognizing VSIG1 as a gastric-enriched differentiation marker shifts its use from defining tumor origin toward refining phenotypic characterization. This distinction influences diagnostic panel construction, reduces the risk of erroneous site attribution in complex cases, and opens avenues for research into epithelial identity and lineage plasticity in gastrointestinal tumors. In this context, the aim of this narrative review is to reframe VSIG1 beyond its traditional classification as a gastric marker and to explore its relevance within the wider context of digestive tract tumor biology. By integrating current knowledge on VSIG1 structure, expression patterns, and biological implications, this review seeks to position VSIG1 as a context-dependent indicator of epithelial differentiation and phenotypic stability rather than a universal oncologic biomarker. Particular emphasis is placed on conceptual interpretation, clinical relevance, and the limitations that currently shape our understanding of VSIG1 in gastrointestinal neoplasia.

## 2. Materials and Methods

### 2.1. Study Design

This work was conducted as a concept-driven narrative review of the literature focusing on the biological, pathological, and clinical relevance of VSIG1 in digestive tract neoplasms. While not designed as a systematic review or meta-analysis, a structured and transparent literature search strategy was applied to ensure comprehensive coverage of the available evidence and to minimize selection bias.

### 2.2. Literature Search Strategy

A comprehensive literature search was performed in the PubMed/MEDLINE, Web of Science Core Collection, and Scopus databases. The search covered studies published from January 2000 to December 2024. Although the search period started in 2000, studies meeting the inclusion criteria and providing clinically relevant data on VSIG1 expression in gastrointestinal neoplasia were identified predominantly after 2014. Earlier publications either lacked tumor-related data or did not address VSIG1 in a clinically interpretable context. Search queries were constructed using combinations of terms related to VSIG1 and gastrointestinal tumors. The primary search terms included: “VSIG1” or “V-set and immunoglobulin domain containing 1”, “gastric cancer”, “stomach cancer”, “gastrointestinal cancer” or “digestive system neoplasms”, “epithelial differentiation” or “lineage marker”, and “immunohistochemistry” or “tumor phenotype”. The full database-specific search syntax, including Boolean operators, field tags, and applied filters, is detailed in Supplementary Table S1 to enhance methodological transparency and allow reproducibility.

### 2.3. Eligibility Criteria

Studies were considered eligible for inclusion if they met the following criteria: original research articles, reviews, or short communications addressing VSIG1 expression, regulation, or function, studies involving human tissues, human-derived cell lines, or clinically relevant experimental models, publications reporting data on normal gastrointestinal epithelium, gastric cancer, or non-gastric digestive neoplasms, including esophageal, colorectal, and hepatobiliary tumors, articles published in English. Conference abstracts, editorials without original data, and studies mentioning VSIG1 only within large gene expression lists without specific data on protein expression, localization, or functional relevance were excluded.

### 2.4. Study Selection and Data Extraction

**Figure 1:**
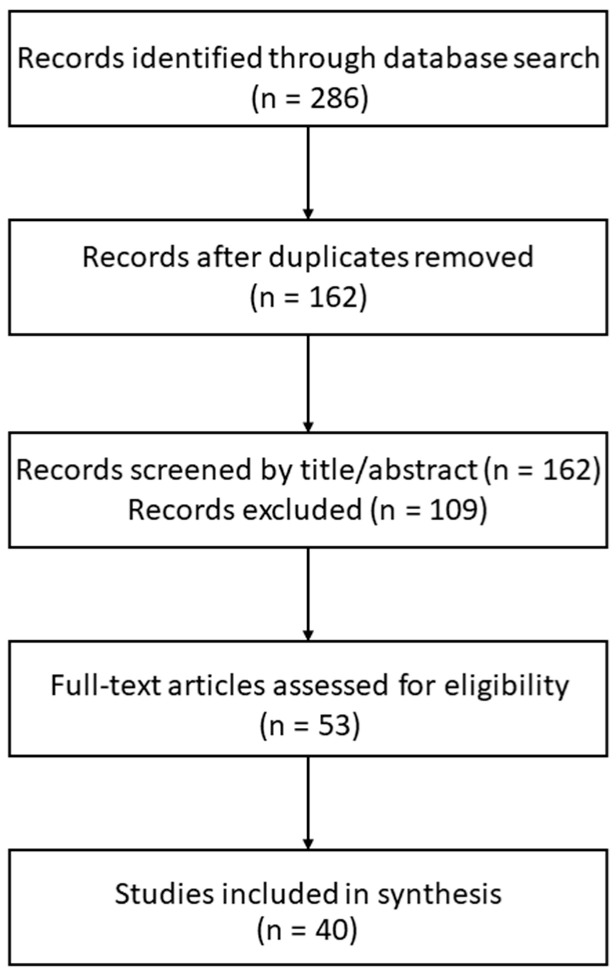
Simplified PRISMA flow diagram of study selection. Flowchart illustrating the identification, screening, eligibility assessment, and inclusion of studies evaluating VSIG1 expression in gastrointestinal tumors and other tissues. A total of 286 records were identified across three databases; after duplicate removal and screening, 40 studies were included in the qualitative synthesis.

Titles and abstracts retrieved from the initial search were screened for relevance. Full-text articles were subsequently reviewed when VSIG1 was explicitly addressed in relation to epithelial differentiation, tumor phenotype, or gastrointestinal pathology. From each included study, the following information was extracted where applicable: study design, tumor type, tissue or model system analyzed, method of VSIG1 assessment, reported expression patterns, and main biological or clinical conclusions. Due to the narrative nature of this review and the heterogeneity of the available data, no quantitative synthesis or meta-analytic approach was undertaken. The initial database search retrieved 96 records from PubMed/MEDLINE, 78 from Web of Science, and 112 from Scopus (total *n* = 286). After removal of duplicates (*n* = 124), 162 unique records remained. Title and abstract screening excluded 109 articles that did not meet eligibility criteria. Full-text assessment was performed for 53 articles, of which 40 studies were ultimately included in the qualitative synthesis. Screening and data extraction were performed independently by two authors (C.-B.S. and A.-M.G.), with discrepancies resolved through discussion and consensus. No formal inter-reviewer agreement statistics were calculated due to the narrative nature of the review (Figure 1).

### 2.5. Data Synthesis and Conceptual Framework

The primary conceptual axes guiding this synthesis included: (1) normal versus neoplastic expression patterns, (2) degree of epithelial differentiation, and (3) anatomic site and lineage context. Emphasis was placed on identifying recurring biological themes, patterns of differentiation-associated expression, and context-dependent interpretations of VSIG1 across gastrointestinal neoplasms. Particular attention was given to discrepancies between studies, limitations of experimental approaches, and gaps in current knowledge, with the aim of framing VSIG1 within a broader conceptual model of epithelial lineage commitment and phenotypic plasticity in cancer. Artificial intelligence–assisted tools (ChatGPT-5.3, OpenAI, San Francisco, CA, USA) were used for language refinement and for generating a conceptual schematic. The figure represents a conceptual illustration synthesized from published literature and does not contain primary or derived experimental data. All AI-assisted outputs were subsequently reviewed, verified against the source literature, and edited by the authors to ensure scientific accuracy, and the authors take full responsibility for the final version of the manuscript.

## 3. VSIG1 in the Context of the Immunoglobulin Superfamily

VSIG1 belongs to the immunoglobulin superfamily (IgSF), a large and functionally diverse group of proteins characterized by the presence of immunoglobulin-like domains [11]. Members of this family participate in a wide range of biological processes, including cell–cell adhesion, maintenance of tissue architecture, immune regulation, and signal transduction [12]. In epithelial tissues, IgSF proteins are frequently involved in the establishment and preservation of polarity, intercellular junctions, and lineage-specific organization [13]. From a structural perspective, VSIG1 contains a V-set immunoglobulin domain, placing it among Ig family members that are often involved in adhesive or recognition-related functions rather than classical immune signaling. While the precise binding partners and downstream pathways of VSIG1 remain incompletely characterized, its molecular architecture suggests a role that is more closely aligned with epithelial organization than with immune modulation. This distinction is important, as it frames VSIG1 as a component of tissue identity rather than as a mediator of inflammatory or immune-driven tumor processes [7,10].

**Figure 2:**
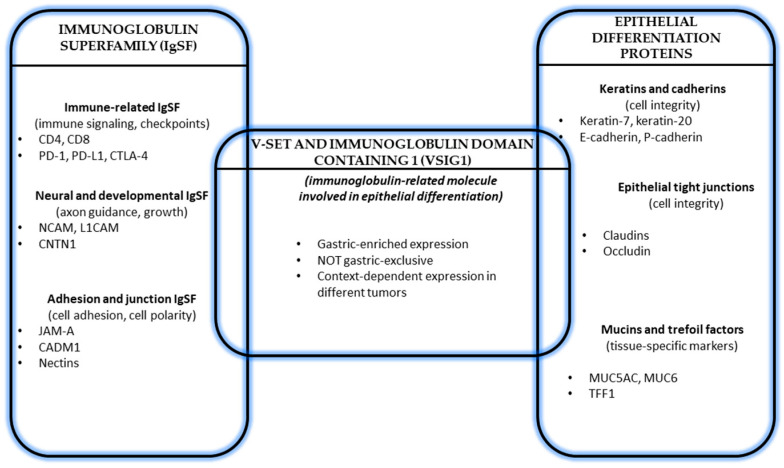
VSIG1 at the interface between the immunoglobulin superfamily (IgSF) and epithelial differentiation proteins. VSIG1 displays gastric-enriched, context-dependent expression, linking Ig-related molecules with epithelial differentiation states. Molecules and functional categories depicted in this schematic are based on published data regarding IgSF proteins involved in epithelial adhesion and differentiation [11,12,13,14].

Within the IgSF, several proteins exhibit tissue-restricted expression patterns and function as markers of epithelial differentiation [14]. Such proteins tend to be tightly regulated during development and maintained in adult tissues as part of stable differentiation programs. Disruption of their expression is commonly observed in malignancy and often reflects loss of epithelial identity rather than direct oncogenic activation [2]. VSIG1 appears to fit within this subgroup, given its selective expression in normal tissues and its consistent association with differentiated epithelial phenotypes [8]. The relevance of IgSF membership also extends to how VSIG1 expression is interpreted in neoplastic settings. In cancer biology, proteins belonging to adhesion-related families are frequently repurposed during tumor progression, either through downregulation, aberrant localization, or context-dependent re-expression [15]. For differentiation-associated Ig proteins, loss of expression often parallels dedifferentiation and phenotypic instability, whereas retained expression may indicate preservation of lineage traits [2]. Understanding VSIG1 through this lens helps explain why its expression patterns in tumors are more informative about differentiation status than about proliferative or mutational burden [16]. Although VSIG1 shows enriched expression in gastric epithelium, available data indicate that it is not exclusively restricted to the gastric mucosa, and its expression should be interpreted in relation to epithelial differentiation state rather than tissue specificity [7] (Figure 2).

Importantly, the structural classification of VSIG1 does not imply functional equivalence with other IgSF members commonly studied in oncology [11]. While immune checkpoint molecules and adhesion receptors have attracted significant attention as therapeutic targets, VSIG1 occupies a distinct conceptual space [17]. Its apparent lack of widespread immune involvement and its association with epithelial differentiation suggest that its primary value lies in biological interpretation and diagnostic context rather than in direct therapeutic exploitation [15]. Placing VSIG1 within the IgSF, therefore, provides a useful biological model without overextending functional assumptions. It highlights the likelihood that VSIG1 participates in epithelial organization and lineage maintenance, while simultaneously emphasizing the limitations of current knowledge regarding its precise molecular interactions [14]. This perspective supports a cautious but biologically grounded interpretation of VSIG1 expression in digestive tract neoplasia, setting the stage for subsequent discussion of tissue-specific expression patterns and differentiation-related dysregulation [18].

## 4. VSIG1 Expression and Epithelial Differentiation

Tissue-enriched gene expression has gained increasing attention in epithelial cancer research, particularly in studies addressing differentiation and lineage identity [19]. Proteins that display preferential expression in specific epithelial compartments are often linked to stable differentiation programs rather than to general cellular functions. In contrast to ubiquitously expressed molecules, such proteins tend to reflect epithelial specialization and are therefore sensitive to changes occurring during malignant transformation [20].

In epithelial cancers, alterations in tissue-enriched expression patterns are common and frequently accompany tumor progression [2]. Multiple studies have shown that markers associated with normal epithelial differentiation are more often retained in well-differentiated tumors and progressively reduced or lost in poorly differentiated carcinomas [8,10,21]. These changes are generally interpreted as part of a broader process of dedifferentiation, rather than as isolated molecular events [22]. Importantly, this phenomenon has been documented across different gastrointestinal tumor types, supporting its relevance as a general biological principle [16]. VSIG1 can be viewed within this framework of tissue-enriched epithelial proteins. Physiological expression studies consistently indicate that VSIG1 shows its highest and most stable expression levels in gastric epithelium [7]. However, available data also demonstrate that VSIG1 is not strictly gastric-specific [23]. Low-level VSIG1 expression outside the stomach has been reported primarily in testis, esophagus and lung, at the transcript level, in limited epithelial compartments identified through large-scale datasets such as The Human Protein Atlas [7,19,23]. Protein-level evidence based on immunohistochemistry indicates predominant gastric expression, whereas transcriptomic datasets show minimal expression in selected non-gastric tissues. These findings support the classification of VSIG1 as gastric-enriched rather than strictly gastric-restricted. This distribution pattern supports the classification of VSIG1 as a gastric-enriched, rather than gastric-restricted, protein [19].

The distinction between tissue enrichment and tissue exclusivity is biologically relevant. Proteins with enriched expression profiles are often embedded in dominant differentiation programs while remaining permissive to low-level expression elsewhere [19,24]. In this respect, VSIG1 differs from broadly expressed adhesion or junctional proteins, yet does not fulfil criteria for absolute tissue specificity [7]. Its expression pattern instead suggests preferential involvement in gastric epithelial differentiation, with potential secondary roles in other epithelial contexts. In gastric cancer, several independent studies have reported an association between VSIG1 expression and tumor differentiation. VSIG1 positivity is more frequently observed in well- and moderately differentiated adenocarcinomas, whereas reduced or absent expression is more common in poorly differentiated tumors [10]. In addition, intratumoral heterogeneity of VSIG1 expression has been described, with preservation in areas maintaining glandular architecture and loss in regions showing structural disorganization. Such findings reinforce the link between VSIG1 expression and local differentiation status rather than overall tumor burden [10,25].

These observations align with broader models of epithelial tumor progression, in which loss of differentiation-associated markers reflects progressive attenuation of lineage identity [2]. Importantly, this process is neither uniform nor linear. Tumors may retain partial differentiation programs or re-express lineage-associated markers in specific contexts. As a result, expression of tissue-enriched proteins such as VSIG1 should be interpreted dynamically, taking into account tumor heterogeneity and histological context [10,22,25].

The biological implications of tissue-enriched expression become more complex when VSIG1 is detected outside the stomach. In non-gastric digestive tumors, VSIG1 expression is uncommon but reproducible [7,10,25]. When present, it is typically associated with tumors exhibiting gastric-type, mixed, or aberrant differentiation patterns. Similar behavior has been reported for gastric lineage-associated proteins such as MUC5AC and TFF1, which may be focally expressed in non-gastric tumors exhibiting gastric-type differentiation, supporting the interpretation that VSIG1 expression in these settings reflects partial activation of gastric differentiation programs rather than nonspecific expression or true lineage conversion [24,25].

From a diagnostic perspective, tissue-enriched markers provide valuable contextual information but have clear limitations [26]. VSIG1 expression should not be interpreted in isolation and must be evaluated alongside histological features and other differentiation markers. Absence of VSIG1 does not exclude gastric origin, particularly in poorly differentiated tumors, while its presence in non-gastric sites requires careful phenotypic correlation [21,26]. These constraints emphasize the importance of using VSIG1 as part of an integrated diagnostic approach rather than as a stand-alone indicator [25,27]. Overall, tissue-enriched expression represents a biologically informative signal that reflects epithelial identity and differentiation status [19]. In the case of VSIG1, its preferential expression in gastric epithelium, combined with its variable presence in other tissues and tumors, supports its role as a marker of lineage stability rather than a universal biomarker of malignancy. Recognizing this distinction is essential for accurate biological interpretation and for avoiding overstatement of its diagnostic or prognostic value in digestive tract neoplasms [10,25]. Expanded diagnostic considerations and practical guidelines are detailed in Section 10.

## 5. Gastric Epithelium as the Biological Reference Point

The gastric epithelium provides a useful biological reference for understanding VSIG1 expression because it represents the tissue in which VSIG1 shows its most consistent and highest physiological expression [7]. Unlike many gastrointestinal markers that are shared across multiple epithelial compartments, VSIG1 expression in normal tissues appears closely aligned with gastric epithelial differentiation, making the stomach a logical starting point for biological interpretation. Normal gastric mucosa is characterized by a well-organized epithelial architecture with region-specific differentiation programs. These programs regulate not only secretory function but also cellular polarity, glandular organization, and intercellular cohesion. Markers associated with gastric differentiation tend to reflect these structural features and are often downregulated when epithelial organization is disrupted [21]. In this setting, VSIG1 expression has been reported to parallel the presence of differentiated gastric epithelial structures rather than simple epithelial presence [10].

Several studies examining normal gastric tissue have shown that VSIG1 expression is most prominent in differentiated epithelial compartments, supporting its association with mature epithelial states [7,9,10]. While detailed functional data remain limited, this expression pattern suggests preferential association with mature epithelial states, although direct data regarding progenitor or stem-like compartments remain limited [28]. This distinction is important, as loss of mature differentiation markers is a recurring feature in gastric carcinogenesis [29]. The relevance of gastric epithelium as a reference point becomes more apparent when considering the heterogeneity of gastric cancer. Gastric adenocarcinoma encompasses a wide spectrum of histological patterns, ranging from well-formed glandular structures to poorly differentiated or diffuse growth patterns [21,30]. Within this spectrum, differentiation-associated markers provide insight into how closely tumor cells resemble their tissue of origin. VSIG1 expression has been shown to follow this gradient, with higher expression in tumors retaining glandular architecture and reduced expression in tumors showing loss of epithelial organization [10]. Importantly, the gastric reference point also helps contextualize variability in VSIG1 expression across studies. Differences in tumor sampling, histological composition, and intratumoral heterogeneity can influence reported expression rates. Studies that specifically correlate VSIG1 expression with histological differentiation tend to show more consistent patterns than those treating gastric cancer as a homogeneous entity. For example, Chen et al. and Satala et al. specifically correlated VSIG1 expression with histological differentiation and reported more consistent associations when architectural features were considered. This observation underscores the need to interpret VSIG1 expression within a defined morphological and biological framework [8,10,25].

The gastric epithelium also serves as a reference point for interpreting VSIG1 expression in non-gastric contexts. When VSIG1 is detected in tumors arising outside the stomach, comparison with its expression pattern in normal gastric mucosa can provide clues regarding the underlying differentiation program [31]. Rather than implying gastric origin per se, such expression may indicate partial activation of gastric-type differentiation pathways, a phenomenon increasingly recognized in gastrointestinal tumor biology. Confirmation of gastric-type differentiation in such settings requires integration with mucin profiles and additional lineage markers, as VSIG1 alone does not define this phenotype [25,31,32]. At the same time, it is important to avoid overextending the gastric reference model. VSIG1 expression alone cannot define epithelial identity, nor does it capture the full complexity of gastric differentiation. Other markers and molecular features contribute to epithelial specification, and VSIG1 should be viewed as one component within a broader differentiation landscape [32]. Recognizing these limitations is essential for avoiding overly simplistic interpretations. In routine diagnostic practice, gastric-type differentiation is commonly supported by expression of markers such as MUC5AC and MUC6 (foveolar and pyloric gland mucins), trefoil factors (TFF1, TFF2), and, in appropriate contexts, markers reflecting gastric lineage commitment. VSIG1 does not duplicate these markers but rather complements them by reflecting epithelial organization and differentiation status rather than mucin production alone [25]. A practical reference panel in tumors with suspected gastric-type differentiation may therefore include VSIG1 alongside representative mucins and site-specific lineage markers, allowing distinction between preserved gastric-enriched programs and aberrant or mixed phenotypes.

In summary, the gastric epithelium represents a biologically grounded reference point for understanding VSIG1 expression. Its preferential expression in normal gastric mucosa and its association with differentiated epithelial architecture provide a framework for interpreting changes observed in gastric cancer and beyond. This reference-based approach allows VSIG1 to be integrated into models of epithelial differentiation and tumor progression without attributing to it an exclusivity or functional role that is not supported by current evidence.

## 6. Dysregulation of Differentiation Programs in Gastric Cancer

Disruption of epithelial differentiation programs represents a central event in gastric carcinogenesis. Unlike tumors driven predominantly by single dominant oncogenic pathways, gastric cancer evolves through progressive alterations in epithelial identity, architecture, and lineage commitment [29]. These changes are reflected not only at the genetic level but also at the level of cellular phenotype, where loss of differentiation is frequently associated with increased invasiveness and biological aggressiveness. Gastric adenocarcinoma displays marked heterogeneity with respect to differentiation, both between tumors and within individual lesions. Well-differentiated tumors often retain glandular structures that resemble normal gastric mucosa, whereas poorly differentiated and diffuse-type carcinomas show profound architectural disruption [21,29]. This morphological diversity is mirrored by changes in the expression of differentiation-associated proteins, many of which are downregulated or lost as tumors progress toward less differentiated states [33]. Several studies have demonstrated that markers linked to gastric epithelial differentiation are preferentially expressed in tumors retaining glandular architecture and are progressively diminished in poorly differentiated carcinomas. This pattern suggests that dysregulation of differentiation is not a binary event but rather a gradual process, occurring alongside tumor progression. Importantly, these changes are often spatially heterogeneous, with differentiated and dedifferentiated areas coexisting within the same tumor mass [30].

**Figure 3:**
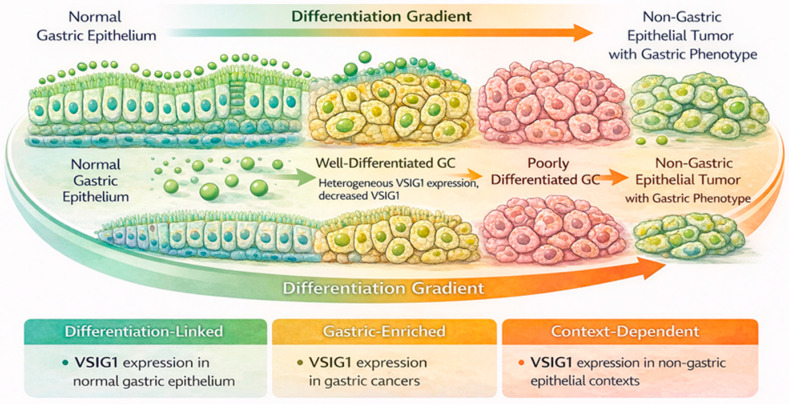
Differentiation-dependent expression of VSIG1 across epithelial states and tumor contexts. Conceptual schematic illustrating the gradient of VSIG1 expression from normal gastric epithelium through well-differentiated and poorly differentiated gastric cancer to non-gastric epithelial tumors with gastric-like features. The diagram summarizes patterns synthesized from published literature [7,10], and does not represent primary experimental data (abbreviations: GC, gastric cancer, VSIG1, V-set and immunoglobulin domain-containing 1).

VSIG1 expression has been reported to follow this differentiation gradient in gastric cancer. For instance, Chen et al. reported VSIG1 expression in approximately 60–70% of well- to moderately differentiated gastric carcinomas compared with markedly lower rates in poorly differentiated tumors, while Satala et al. observed positivity in approximately 50–65% of cases, with higher prevalence in areas retaining glandular architecture [8,10,25]. Rather than being uniformly expressed or lost across all tumor cells, VSIG1 expression appears to correlate with preserved epithelial organization. Areas of tumors that maintain glandular features are more likely to retain VSIG1 expression, whereas regions showing loss of polarity, discohesive growth, or diffuse infiltration frequently exhibit reduced or absent expression [10]. This spatial association reinforces the concept that VSIG1 reflects local differentiation status rather than global tumor behavior (Figure 3).

The dysregulation of differentiation programs in gastric cancer is closely linked to broader biological processes, including epithelial–mesenchymal transition (EMT) and increased phenotypic plasticity. As tumors lose epithelial characteristics, they often acquire features associated with motility and invasion. In this context, downregulation of differentiation-associated proteins is part of a coordinated shift in cellular state rather than an isolated molecular alteration [34]. VSIG1 loss in poorly differentiated tumors is therefore best interpreted as a marker of this transition rather than as a direct contributor to EMT. Importantly, not all poorly differentiated gastric cancers show complete loss of differentiation markers. Partial retention of lineage-associated proteins has been observed in subsets of tumors, suggesting that differentiation programs may be incompletely suppressed or variably reactivated [25]. Such findings highlight the dynamic nature of differentiation dysregulation and caution against overly simplistic models that equate loss of a single marker with complete dedifferentiation.

Epigenetic mechanisms are thought to play a significant role in the dysregulation of differentiation programs in gastric cancer. Changes in DNA methylation, histone modification, and chromatin organization have been shown to affect the expression of lineage-associated genes. While direct mechanistic links between these processes and VSIG1 regulation remain incompletely defined, the observed expression patterns are consistent with epigenetically driven modulation rather than irreversible genetic loss [35,36].

The clinical implications of differentiation dysregulation are complex. Although poorly differentiated gastric cancers are generally associated with worse outcomes, differentiation status alone does not fully capture tumor behavior. Markers such as VSIG1 provide information about epithelial identity but do not directly predict prognosis or therapeutic response [7]. Their value lies in contextualizing tumor phenotype rather than serving as independent clinical endpoints. Taken together, dysregulation of differentiation programs represents a fundamental aspect of gastric cancer biology. VSIG1 expression patterns align closely with this process, reflecting preservation or loss of epithelial identity [10,25]. Understanding VSIG1 within this broader framework allows its expression to be interpreted as part of a dynamic differentiation landscape rather than as an isolated molecular event. This perspective provides a basis for subsequent discussion of VSIG1 behavior in non-gastric digestive tract tumors and in contexts of aberrant lineage commitment.

## 7. VSIG1 Expression in Gastric Cancer: Patterns, Variability, and Interpretation

Studies assessing VSIG1 expression in gastric cancer describe a wide range of expression patterns rather than a uniform presence or absence across tumors [10]. This variability is consistently reported and represents a biological feature rather than a methodological artifact. Interpretation of VSIG1 expression, therefore, requires attention to histological context, tumor architecture, and sampling strategy. At the level of intertumoral variability, VSIG1 expression is more frequently detected in well- and moderately differentiated gastric adenocarcinomas. Tumors retaining glandular structures and epithelial cohesion are more likely to show VSIG1 positivity, whereas poorly differentiated carcinomas and diffuse-type tumors often lack detectable expression. These associations are more evident in intestinal-type tumors according to Lauren classification, whereas diffuse-type carcinomas more frequently lack VSIG1 expression [8,10]. These associations have been reported across independent cohorts, supporting the link between VSIG1 expression and differentiation status rather than tumor subtype alone [7,10]. Intratumoral heterogeneity represents one of the most reproducible findings related to VSIG1 expression. Several studies describe patchy or focal staining patterns within the same tumor, with VSIG1-positive regions corresponding to areas of preserved glandular organization. In contrast, adjacent regions displaying discohesive growth, loss of polarity, or infiltrative patterns frequently show reduced or absent expression. This spatial variability indicates that VSIG1 reflects local epithelial differentiation rather than global tumor classification [7,10,25].

Sampling-related factors further contribute to variability in reported expression rates. Small biopsies or tissue microarrays may underrepresent differentiated tumor components, leading to apparent absence of VSIG1 in cases where expression is actually present focally. Studies that correlate immunohistochemical findings with whole-section histology tend to report more consistent associations between VSIG1 expression and morphological features, emphasizing the importance of adequate tissue evaluation. Differences in subcellular localization have also been noted across studies. Membranous staining patterns are more commonly observed in well-organized epithelial structures, whereas cytoplasmic or weak diffuse staining has been reported in less differentiated tumor areas [10]. The biological significance of these localization patterns remains uncertain. They may reflect alterations in protein trafficking, changes in cell polarity, or technical differences related to antibody performance and tissue processing. Regardless of the underlying mechanism, variability in localization further complicates binary scoring approaches.

Methodological heterogeneity across studies represents an additional source of variation. Differences in antibodies, scoring criteria, and cut-off definitions make direct comparison challenging. Studies that dichotomize VSIG1 expression without reference to differentiation or architectural features often report weaker associations, whereas analyses incorporating morphological context provide more informative results. This highlights the risk of oversimplification when interpreting VSIG1 expression in gastric cancer [10,34]. Temporal aspects of tumor evolution may also influence observed expression patterns. Gastric cancer progression is associated with dynamic changes in differentiation state, and it is plausible that VSIG1 expression fluctuates during this process. Although longitudinal data are limited, the presence of intratumoral heterogeneity supports the notion that VSIG1 expression is not fixed but may change as tumors evolve or respond to treatment [7,10].

Importantly, VSIG1 expression does not show a consistent independent association with clinical outcome across studies. While poorly differentiated tumors generally have worse prognosis, VSIG1 status alone does not reliably stratify patients in prognostic terms [7]. This finding reinforces the interpretation of VSIG1 as a differentiation-associated marker rather than a surrogate for tumor aggressiveness or stage. The collective evidence indicates that VSIG1 expression in gastric cancer is best understood as a reflection of epithelial organization and differentiation at both intertumoral and intratumoral levels. Variability in expression should therefore be expected and interpreted in light of tumor architecture, sampling, and methodological design [25]. Failure to account for these factors risks misclassification and overinterpretation of VSIG1 status. These considerations are particularly relevant when extending interpretation beyond gastric cancer. The context-dependent behavior of VSIG1 observed in gastric tumors provides a framework for understanding its expression in non-gastric digestive tract neoplasms, where differentiation programs are often aberrant or incomplete rather than absent.

## 8. VSIG1 Expression Beyond the Stomach: Non-Gastric Digestive Tumors and the Hepato-Gastric Differentiation Axis

Evaluation of VSIG1 expression outside the stomach has revealed a pattern that is uncommon but consistent in that expression aligns with activation of gastric-type differentiation programs rather than random distribution across tumor types. Across non-gastric digestive tract tumors, VSIG1 positivity is generally infrequent and rarely diffuse [31,36]. When present, it tends to localize to tumors or tumor components that display gastric-type or mixed differentiation rather than reflecting the tumor site alone. This distribution supports the interpretation of VSIG1 as a marker linked to activation of gastric-enriched differentiation programs rather than as an indicator of anatomical origin [31].

In colorectal, gastroesophageal junction, and pancreatic tumors, VSIG1 expression has been reported primarily in association with gastric-type phenotypes [7]. These include tumors with mucin profiles resembling gastric epithelium, glandular architectures deviating from site-specific norms, or areas showing partial loss of canonical intestinal differentiation. In such settings, VSIG1 expression is typically focal and heterogeneous, mirroring the patchy activation of alternative differentiation programs within an otherwise site-appropriate tumor background. This focal expression pattern highlights an important conceptual point: VSIG1 does not define a stable lineage state in non-gastric tumors but rather marks partial or incomplete engagement of gastric differentiation pathways [10,31]. As such, VSIG1 behaves similarly to other lineage-associated proteins that are re-expressed in aberrant contexts during tumor evolution. Its expression reflects phenotypic plasticity rather than lineage conversion, and its absence in most non-gastric tumors emphasizes its specificity for gastric-enriched programs.

The biological logic underlying this pattern becomes more apparent when considering tumors with hepato-gastric phenotypic overlap. Hepatoid gastric adenocarcinoma and hepatocellular carcinoma with gastric-type features represent two ends of a spectrum in which differentiation programs characteristic of gastric epithelium and hepatocytes intersect [10,31]. In these tumors, VSIG1 expression has been documented in subsets of cases, particularly in components retaining glandular or gastric-type differentiation, despite the presence of hepatocytic morphology or hepatic marker expression. In hepatoid gastric adenocarcinoma, preservation of VSIG1 expression in certain tumor areas supports the notion that gastric differentiation programs may persist even as tumors acquire hepatocytic features. Conversely, in hepatocellular carcinomas exhibiting gastric-type differentiation, VSIG1 expression appears to reflect partial activation of gastric epithelial programs rather than misclassification of tumor origin [31,32]. In both scenarios, VSIG1 expression aligns more closely with differentiation state than with morphology or anatomical site.

These observations point toward a shared foregut differentiation axis linking gastric and hepatic lineages. Both tissues arise from foregut endoderm, and it is increasingly recognized that tumors originating from this region may access overlapping transcriptional and differentiation programs under pathological conditions [10]. Within this framework, VSIG1 can be viewed as an indicator of gastric-enriched differentiation activity along this axis, independent of whether the tumor arises in the stomach, liver, or another gastrointestinal site [7,10,31,32]. From a diagnostic perspective, this integrated view has practical implications. VSIG1 expression outside the stomach should prompt consideration of gastric-type differentiation or aberrant lineage engagement rather than immediate assumptions regarding tumor origin. In hepatoid tumors, VSIG1 positivity supports retention or reactivation of gastric programs but does not negate hepatocytic differentiation. Interpretation, therefore, requires correlation with morphology, mucin phenotype, and a broader immunohistochemical panel [25].

Biologically, available data do not support a direct functional role for VSIG1 in driving hepatoid or gastric differentiation. Instead, its expression appears to mark the state of differentiation programs that are variably activated during tumor progression. This behavior is consistent with the broader pattern observed across gastrointestinal neoplasms, where VSIG1 expression tracks differentiation context and phenotypic plasticity rather than oncogenic potential. Viewed together, VSIG1 expression in non-gastric digestive tract tumors overlap reinforces its role as a marker of gastric-enriched differentiation operating across tissue boundaries. Recognition of this shared framework allows VSIG1 to be interpreted coherently across diverse tumor types and emphasizes the limitations of rigid organ-based classification in the face of tumor plasticity.

From a practical standpoint, evaluation of VSIG1 in hepatoid or hepato-gastric overlap tumors should follow a structured panel-based approach: (1) In hepatoid gastric carcinoma: assess VSIG1 together with gastric-type mucins (MUC5AC/MUC6) and hepatic markers (HepPar-1, AFP). VSIG1 positivity in glandular areas supports retained gastric differentiation despite hepatocytic morphology. (2) In hepatocellular carcinoma with gastric-type features: VSIG1 should be interpreted alongside canonical hepatic markers (HepPar-1, Arginase-1) and clinical–radiological correlation. Focal VSIG1 expression indicates activation of gastric-enriched differentiation programs and does not in itself imply metastatic gastric origin. This structured interpretation reduces the risk of over-attributing tissue origin based on a single marker.

Key studies supporting these observations and illustrating the evolution of VSIG1-related research across gastrointestinal tumors are summarized in Table 1 [7,8,10,25,31,32,36,37].

## 9. VSIG1 and Aberrant Lineage Commitment in Gastrointestinal Neoplasia

Aberrant lineage commitment has emerged as a key concept in the biology of gastrointestinal cancers [38,39]. Rather than maintaining a fixed differentiation state, many tumors display partial, mixed, or unstable lineage programs that evolve during progression. This phenomenon is increasingly recognized across gastrointestinal sites and provides a useful framework for interpreting markers whose expression does not strictly follow anatomical boundaries [2,40]. VSIG1 fits well within this model. Lineage commitment in normal gastrointestinal epithelium is tightly regulated and results in stable, site-specific differentiation programs [7]. In cancer, however, these programs may be incompletely maintained, suppressed, or aberrantly reactivated. Tumor cells may lose features of their tissue of origin while simultaneously acquiring characteristics associated with alternative epithelial lineages. This process does not necessarily imply transdifferentiation in a strict sense, but rather reflects plasticity within a shared developmental or transcriptional landscape. Evidence supporting aberrant lineage commitment in gastrointestinal tumors comes from multiple sources, including mixed histological patterns, discordant immunophenotypes, and overlapping mucin expression profiles [25,30,32]. Tumors may express markers that are not typical for their anatomical site, often in a focal or heterogeneous manner. These observations challenge rigid organ-based classification and suggest that lineage identity in cancer is better understood as a spectrum rather than a binary state.

Within this context, VSIG1 behaves as a marker that reports activation of gastric-enriched differentiation programs. Its expression outside the stomach, including in colorectal, pancreatic, and hepatic tumors with gastric-type features, is difficult to explain with a strict tissue-specific model. Instead, these patterns are more consistent with partial engagement of gastric lineage pathways in tumors that otherwise arise from non-gastric epithelium [31,35]. Aberrant lineage commitment also provides a framework for understanding intratumoral heterogeneity. Tumors often contain subpopulations of cells that differ in differentiation state and lineage marker expression. VSIG1-positive and VSIG1-negative regions within the same tumor may therefore represent distinct phenotypic states rather than clonal differences. This interpretation aligns with observations that VSIG1 expression correlates with preserved epithelial architecture at the local level, even when the tumor as a whole is poorly differentiated [32].

The gastro-hepatic overlap discussed in the previous section illustrates this concept particularly well. Tumors arising from foregut-derived tissues appear capable of activating overlapping differentiation programs under pathological conditions. VSIG1 expression in this setting does not signal a change in tumor origin but rather reflects access to a shared lineage repertoire [10,25,31]. Such behavior underscores the limitations of interpreting lineage markers solely through the lens of tissue origin. Importantly, aberrant lineage commitment is not a uniform or obligatory feature of tumor progression. Many gastrointestinal cancers retain relatively stable differentiation programs throughout their course. VSIG1 expression is therefore not expected in all tumors, nor does its absence indicate a lack of plasticity. Instead, VSIG1 identifies a subset of tumors or tumor components in which gastric-enriched programs are active or partially reactivated [31,35].

From a biological standpoint, there is currently no evidence that VSIG1 initiates or drives aberrant lineage commitment. Its expression appears to be downstream of broader transcriptional and epigenetic changes that govern differentiation state. As such, VSIG1 should be viewed as a marker of lineage engagement rather than a regulator of lineage choice. This distinction is important for avoiding overinterpretation of its functional role.

Recognition of aberrant lineage commitment has practical implications for pathology and tumor classification. Markers such as VSIG1 are most informative when interpreted as indicators of phenotypic state rather than determinants of origin. In diagnostically challenging cases, their expression can highlight mixed or atypical differentiation patterns, prompting more nuanced classification and appropriate use of complementary markers [10]. Framing VSIG1 within the concept of aberrant lineage commitment allows its diverse expression patterns across gastrointestinal neoplasia to be interpreted coherently. Rather than representing exceptions or anomalies, non-gastric expression of VSIG1 reflects the inherent plasticity of digestive tract tumors. This perspective integrates VSIG1 into a broader model of tumor differentiation and sets the stage for discussing its diagnostic value and limitations in clinical practice.

## 10. Diagnostic Implications of VSIG1 Expression: Utility and Pitfalls

The diagnostic value of VSIG1 lies primarily in its ability to inform on epithelial differentiation rather than to establish tumor origin or biological behavior in isolation. As a marker associated with gastric-enriched differentiation programs, VSIG1 can contribute to the characterization of tumor phenotype when used within a broader immunohistochemical and morphological framework. Its interpretation, however, requires careful attention to context and awareness of its limitations [7].

In gastric cancer, VSIG1 expression may support the presence of preserved gastric epithelial differentiation, particularly in tumors with glandular architecture [10]. In this setting, VSIG1 positivity is most informative when it aligns with histological features of differentiation and with the expression of other gastric markers. Conversely, absence of VSIG1 should not be interpreted as evidence against gastric origin, especially in poorly differentiated or diffuse tumors where loss of differentiation-associated markers is common [10,25].

The diagnostic challenges become more pronounced outside the stomach. In non-gastric gastrointestinal tumors, VSIG1 expression is infrequent and typically focal [31,32]. When detected, it should prompt consideration of gastric-type or mixed differentiation rather than immediate reassignment of tumor origin. This is particularly relevant in tumors arising at anatomical transition zones or in cases with ambiguous morphology, where reliance on single markers can lead to misclassification [7,9]. The hepato-gastric overlap represents a diagnostic setting in which VSIG1 expression can be both informative and misleading. In gastric tumors with hepatoid features, VSIG1 positivity supports retention of gastric differentiation despite hepatocytic morphology [10]. In hepatic tumors, however, VSIG1 expression reflects activation of gastric-enriched programs and does not imply metastatic gastric cancer [31]. Failure to recognize this distinction risks diagnostic error, particularly when VSIG1 is interpreted without integration of morphology and a comprehensive marker panel. VSIG1 is therefore best used as part of a differentiation-oriented panel rather than as a stand-alone marker. Its interpretation should be integrated with mucin expression patterns, markers of intestinal differentiation, and site-appropriate lineage markers [25]. This panel-based approach allows VSIG1 to contribute meaningfully to phenotypic assessment while minimizing the risk of overinterpretation.

Several technical factors further influence diagnostic utility. Variability in antibody performance, scoring thresholds, and evaluation of subcellular localization can affect reported results. Focal expression patterns and intratumoral heterogeneity increase the risk of false-negative interpretations in small biopsies or tissue microarrays [25]. Whole-section analysis and correlation with histological features remain essential for reliable assessment.

Another important limitation is the lack of standardized scoring systems for VSIG1. Most studies report qualitative or semi-quantitative findings, often without clearly defined cut-offs. This variability complicates inter-study comparison and limits immediate clinical translation. Until standardized methodologies are established, VSIG1 should be regarded as a supportive rather than decisive diagnostic marker.

Finally, VSIG1 expression does not provide direct prognostic or predictive information. Its diagnostic value lies in clarifying differentiation status and lineage engagement rather than in stratifying clinical risk or guiding therapy. Attempts to extend its interpretation beyond this role are not supported by current evidence and may obscure its actual utility. In practice, VSIG1 is most useful when interpreted conservatively and contextually. When used to highlight gastric-enriched differentiation within complex or ambiguous tumors, it can add meaningful information. When used in isolation or outside a clear morphological framework, its diagnostic value diminishes. Recognizing these boundaries is essential for integrating VSIG1 into routine diagnostic practice without overstating its significance.

Based on currently available evidence, a minimal practical framework for VSIG1 application may include:

- Indications: tumors with ambiguous gastric-type morphology, suspected mixed differentiation, or hepatoid features.
- Panel context:
  – In primary gastric tumors, VSIG1 should be interpreted alongside gastric-type mucins (MUC5AC, MUC6) and correlated with architectural differentiation.
  – In non-gastric digestive tract tumors (e.g., colorectal or pancreatic carcinomas) with suspected gastric-type differentiation, VSIG1 may be combined with MUC5AC/MUC6, CDX2, and CK7/CK20 pattern assessment to distinguish partial gastric program activation from preserved site-specific differentiation.
  – In hepatoid or hepato-gastric overlap contexts, VSIG1 should be evaluated together with hepatic markers such as HepPar-1 and Arginase-1, and interpreted in conjunction with morphology and clinical–radiological data.
- Technical considerations: whole-section analysis is preferable when feasible, given the frequent focal and heterogeneous staining patterns. In small biopsies and tissue microarrays, negative results should be interpreted cautiously.
- Reporting format: percentage of positive tumor cells, staining intensity (weak/moderate/strong), subcellular localization (membranous vs. cytoplasmic), and presence of intratumoral heterogeneity should be documented.

This structured approach aligns VSIG1 with differentiation-oriented interpretation and minimizes overdiagnosis based on isolated staining results.

## 11. Functional Insights into VSIG1 Biology

Functional data on VSIG1 remain limited when compared with descriptive and clinicopathological studies. Most of the current understanding of VSIG1 biology derives from expression analyses rather than from direct mechanistic investigations. This imbalance has shaped the way VSIG1 is interpreted in the literature, with its role inferred primarily from correlation with differentiation states rather than from demonstrated functional effects [7,8]. Available experimental studies suggest that VSIG1 may be involved in maintaining epithelial characteristics. In cellular models where VSIG1 expression has been manipulated, re-expression has been associated with reduced proliferative and migratory capacity, as well as partial restoration of epithelial features [7]. These observations are consistent with the broader behavior of differentiation-associated proteins, which tend to support epithelial organization rather than promote oncogenic signaling. However, such findings are context-dependent and derived from a limited number of experimental systems.

At the molecular level, the precise functional partners of VSIG1 are not well defined. Its classification within the IgSF and the presence of a V-set domain suggest a potential role in cell–cell interactions or adhesion-related processes [36,37]. Nevertheless, no definitive ligand–receptor interactions or downstream signaling pathways have been conclusively established. As a result, mechanistic interpretations remain cautious and largely inferential. One recurring hypothesis is that VSIG1 contributes indirectly to epithelial polarity or junctional stability. This idea is supported by the observation that VSIG1 expression correlates with preserved glandular architecture and membranous localization in differentiated epithelial cells [36]. Loss of VSIG1 expression in poorly differentiated tumors may therefore reflect disruption of epithelial organization rather than loss of a specific signaling function [7,37]. Experimental validation of this hypothesis, however, is still lacking.

Regulation of *VSIG1* expression also remains incompletely understood. Genetic alterations affecting the *VSIG1* locus have not been consistently reported in gastrointestinal cancers, suggesting that transcriptional or epigenetic mechanisms are more likely to underlie changes in expression. Epigenetic silencing during dedifferentiation, altered transcription factor activity, or broader chromatin remodeling associated with lineage plasticity may all contribute to *VSIG1* downregulation. Direct evidence for these mechanisms is currently sparse. The relationship between *VSIG1* and EMT has been explored in a limited number of studies. These reports suggest that loss of *VSIG1* expression accompanies EMT-like changes rather than initiating them. In this model, *VSIG1* functions as a marker of epithelial state, with its downregulation occurring as part of a coordinated shift toward a more plastic or mesenchymal phenotype [10,32]. This interpretation is consistent with the absence of data supporting a direct regulatory role for *VSIG1* in EMT signaling pathways.

Importantly, the lack of comprehensive mechanistic data does not diminish the biological relevance of *VSIG1*. Instead, it highlights a gap between descriptive pathology and functional biology. *VSIG1* appears to sit downstream of differentiation programs that are altered during tumor progression, making it a useful indicator of epithelial state even in the absence of a clearly defined molecular function. Future mechanistic studies will need to address several open questions, including the identification of *VSIG1* interaction partners, clarification of its subcellular trafficking, and characterization of the regulatory elements controlling its expression. Functional experiments using well-defined epithelial models and lineage-specific contexts will be particularly important for disentangling correlation from causation.

At present, VSIG1 should be viewed as a biologically informative but mechanistically underexplored protein. Its consistent association with differentiation states across gastrointestinal neoplasia supports its relevance, while the absence of definitive functional data cautions against attributing to it roles that extend beyond current evidence.

Given its IgSF V-set domain structure and predominant membranous localization in differentiated epithelial cells, a plausible hypothesis is that VSIG1 contributes indirectly to maintenance of epithelial polarity or junctional stability. Interaction with adhesion-related complexes or cytoskeletal-associated proteins could theoretically stabilize apical–basal polarity and glandular architecture. Under this model, downregulation of VSIG1 would not initiate dedifferentiation but could facilitate architectural disorganization once differentiation programs become unstable. This hypothesis remains speculative and requires experimental validation, but it provides a biologically coherent framework linking VSIG1 structure with its differentiation-associated expression pattern.

## 12. Methodological Limitations and Current Knowledge Gaps

Interpretation of *VSIG1* expression across gastrointestinal neoplasia is constrained by several methodological limitations that recur throughout the literature. These limitations affect not only reported prevalence rates but also the biological and clinical conclusions drawn from available studies. Recognition of these issues is essential for placing existing data into proper context and for guiding future research efforts.

One major limitation is the heterogeneity of study design. Most published analyses of *VSIG1* expression are retrospective and based on relatively small cohorts. Sample sizes vary widely, and inclusion criteria are often not uniform, particularly with respect to tumor subtype, differentiation grade, and anatomical location. As a result, comparisons between studies are difficult, and reported associations may reflect cohort composition rather than biological differences.

Technical variability in immunohistochemical assessment represents another significant source of inconsistency. Different studies have employed distinct antibodies, staining protocols, and scoring systems, often without detailed validation or justification. Cut-off values for defining positivity are rarely standardized, and some reports rely on qualitative descriptions rather than reproducible scoring criteria. This variability complicates interpretation and limits the transferability of findings to routine diagnostic practice. Sampling-related issues further contribute to uncertainty. As discussed in earlier sections, *VSIG1* expression is frequently focal and heterogeneous within tumors. Studies based on tissue microarrays or small biopsies may therefore underestimate expression or misclassify tumors as negative. Whole-section analysis and correlation with histological architecture are more informative but are not consistently applied across studies.

Another limitation is the lack of longitudinal data. Most studies assess *VSIG1* expression at a single time point, typically at initial diagnosis. Changes in expression during tumor progression, recurrence, or treatment are largely unexplored. Given the association between VSIG1 and differentiation state, it is plausible that expression may change over time, but this remains insufficiently documented.

Functional data remain sparse and unevenly distributed across tumor types. While some experimental studies have explored *VSIG1* expression in gastric cancer models, comparable data for non-gastric gastrointestinal tumors and hepato-gastric overlap settings are largely absent. This imbalance limits the ability to generalize observations and to determine whether *VSIG1* behaves similarly across different epithelial contexts. Interpretative bias also deserves consideration. Because VSIG1 has been primarily studied in gastric cancer, its expression in other tumors is often interpreted through a gastric-centric lens. While this approach has heuristic value, it may obscure alternative explanations related to broader epithelial plasticity or shared developmental programs. A more neutral, phenotype-oriented interpretation may be necessary to fully capture the biological significance of VSIG1 expression outside the stomach.

Finally, there is a tendency in some reports to overextend the clinical implications of VSIG1 findings. Associations with differentiation or tumor grade are sometimes presented as evidence of prognostic relevance without adequate multivariate analysis or independent validation. This practice risks inflating expectations regarding clinical utility and underscores the need for cautious interpretation.

Addressing these limitations will require coordinated efforts to standardize analytical approaches, expand cohort sizes, and integrate morphological, immunophenotypic, and molecular data. Until such data are available, conclusions regarding VSIG1 should remain grounded in descriptive and contextual evidence rather than extrapolated beyond what current studies support.

## 13. Future Directions and Research Priorities

Future work on VSIG1 should focus on clarifying its role as a differentiation-associated marker within gastrointestinal and hepatobiliary tumors, rather than attempting to position it as a broad oncologic biomarker. Current data indicate that VSIG1 is most informative when interpreted in relation to epithelial phenotype, tissue context, and tumor architecture [10,25,31]. Research priorities should therefore align with these strengths and address the limitations outlined in previous sections.

One important direction is the standardization of immunohistochemical assessment. Consistent antibody selection, staining protocols, and scoring systems are needed to allow meaningful comparison across studies. Reporting should include not only intensity and extent of staining but also subcellular localization and correlation with histological features. Adoption of such standards would improve reproducibility and facilitate integration of VSIG1 into multi-marker panels used in diagnostic practice.

Larger, well-characterized cohorts are also required. Most existing studies are limited by small sample size and retrospective design. Multicenter analyses with clearly defined inclusion criteria, whole-section evaluation, and detailed histopathological annotation would provide a more reliable picture of VSIG1 expression patterns across tumor types. Particular attention should be given to tumors with mixed or unusual phenotypes, where VSIG1 appears most informative.

Further exploration of VSIG1 expression in non-gastric tumors represents another priority. While preliminary data suggest a role in marking gastric-type differentiation across tissue boundaries, these observations remain fragmentary. Focused studies examining VSIG1 alongside gastric and hepatic markers in such tumors could clarify whether its expression identifies reproducible subgroups with shared phenotypic or biological characteristics.

Experimental studies addressing regulation of VSIG1 expression are also needed. Determining how VSIG1 expression is maintained in differentiated epithelium and lost during dedifferentiation would provide insight into the broader processes governing epithelial identity. Approaches examining transcriptional regulation, chromatin state, and interaction with differentiation-associated transcription factors would be particularly informative. Integration of VSIG1 into multi-parameter analyses represents another promising avenue. Rather than evaluating VSIG1 in isolation, combining its expression with histological features, mucin profiles, and molecular data may yield more informative classifications. Such integrated approaches align with current trends in pathology and could help refine phenotypic stratification in gastrointestinal tumors.

Finally, future studies should maintain realistic expectations regarding clinical application. While VSIG1 contributes valuable information about differentiation state, its role is supportive rather than decisive. Research efforts should therefore aim to define where VSIG1 adds incremental value, rather than attempting to expand its use beyond evidence-supported contexts. Progress in these areas would allow VSIG1 to be more effectively integrated into both research and diagnostic settings, while preserving the nuanced interpretation required by its context-dependent expression.

## 14. Conclusions

VSIG1 should be regarded as a differentiation-associated marker whose significance in gastrointestinal neoplasia is strongly context-dependent. Across studies, its expression consistently aligns with preservation of gastric-enriched epithelial programs, while loss of expression accompanies dedifferentiation and architectural disruption. This behavior supports the interpretation of VSIG1 as an indicator of epithelial identity rather than as a general cancer biomarker.

In gastric cancer, VSIG1 expression reflects differentiation status at both intertumoral and intratumoral levels, frequently showing heterogeneous distribution that mirrors local epithelial organization. Outside the stomach, VSIG1 expression is uncommon but reproducible in tumors exhibiting gastric-type or mixed differentiation, including settings of hepato-gastric phenotypic overlap. In these contexts, VSIG1 marks engagement of gastric-enriched differentiation programs rather than tumor origin. Diagnostic use of VSIG1 is therefore best confined to phenotype-oriented interpretation within multi-marker panels and correlated with morphology. Current data on independent prognostic value are limited and partially conflicting, and predictive roles remain unsupported, while functional data remain limited. Overextension of VSIG1 beyond its role as a differentiation marker risks misinterpretation. Taken as a whole, VSIG1 provides biologically meaningful insight into epithelial differentiation and lineage plasticity across gastrointestinal tumors. Its value lies in refining phenotypic assessment when applied conservatively and in an appropriate biological context. Moving beyond the misconception of strict gastric specificity allows VSIG1 to be repositioned within modern models of tumor classification that emphasize differentiation state and lineage plasticity. This shift enhances diagnostic precision and provides a more biologically coherent framework for future mechanistic research.

## Figures and Tables

**Table 1:** Key Studies Evaluating VSIG1 Expression in Digestive Tract Tumors: Methodological and Immunohistochemical Characteristics.

Year	Authors	Tumor Type(s)	Study Model/Specimen	Antibody/Clone	Specimen Type	Scoring System/Cutoff	Subcellular Localization	Reported Positivity	Key Findings/Interpretation
2006	Scanlan et al. [36]	Normal tissues, epithelial cancers	Human tissue samples, cDNA libraries	Not specified	Tissue samples	Not applicable (gene expression study)	Not specified	Tissue-restricted expression identified	VSIG1 identified as epithelial lineage-associated antigen
2011	Oidovsambuu et al. [37]	Normal gastric epithelium, gastric cancer	Mouse model	Polyclonal antibody	Whole sections	Qualitative assessment	Predominantly membranous	High in glandular gastric epithelium	VSIG1 required for proper gastric epithelial differentiation; differentiation-associated marker rather than proliferation-associated protein
2012	Chen et al. [8]	Gastric cancer	Human tumor samples	Not clearly specified	Whole sections	Semi-quantitative; % positive cells + intensity	Membranous ± cytoplasmic	~60–70% in well/moderately differentiated tumors	Decreased VSIG1 associated with poor differentiation and worse prognosis
2017	Inoue et al. [7]	Gastric, lung, esophageal cancers	Cell lines; human tissues	Not specified	Whole sections	Qualitative	Membranous	Variable; reduced in aggressive phenotypes	Tumor suppressor-like behavior; supports epithelial differentiation
2021	Gurzu et al. [32]	Gastric-type hepatocellular carcinoma	Human tumor samples	Not specified	Whole sections	Semi-quantitative	Membranous	Subset of cases positive (~30–40%)	VSIG1 marks aberrant activation of gastric-type differentiation in hepatic tumors
2022	Satala et al. [10]	Gastric cancer	Human tumor samples	Rabbit polyclonal HPA036311	Tissue microarray	Semi-quantitative (extent + intensity)	Membranous predominance	~50–65%; heterogeneous	Expression correlates with preserved glandular architecture; intratumoral heterogeneity observed
2023	Satala et al. [25]	Gastric adenocarcinoma	Human tumor samples	Rabbit polyclonal HPA036311	Tissue microarray	Correlated with mucin phenotype	Membranous	Higher in gastric-type mucin phenotype	Marker of gastric-enriched epithelial programs
2024	Szodorai et al. [31]	Hepatocellular carcinoma	Human HCC samples	Rabbit polyclonal HPA036311	Whole sections	Semi-quantitative	Focal membranous	Subset of cases positive (~20–35%)	Evidence of gastric-type differentiation signature in hepatic tumors

## Data Availability

The original contributions presented in this study are included in the article. Further inquiries can be directed to the corresponding author.

## Supplementary Materials

The following supporting information can be downloaded at: https://www.mdpi.com/article/10.3390/cancers18050867/s1, Table S1: Database-Specific Search Strategies. Searches were conducted between January 2000 and December 2024. No initial restrictions regarding study design were applied. Reference lists of included articles were manually screened to identify additional relevant publications not captured by database queries.
