# Supplementary material for: Beyond Gastric Specificity: V-Set and Immunoglobulin Domain-Containing 1 (VSIG1) in Digestive Tract Tumors
Source: Cancers (Basel). 2026 Mar 8;18(5):867. doi: 10.3390/cancers18050867 (PMC12984364; doi:10.3390/cancers18050867)
Supplement: Supplementary file 1 [file cancers-18-00867-s001.zip › cancers-4183884-supplementary.pdf]

Database	Search Strategy (Exact Query Syntax)	Filters Applied
**PubMed/MEDLINE**	("VSIG1"[Title/Abstract] OR "V-set and immunoglobulin domain containing 1"[Title/Abstract]) AND ("gastric cancer"[Title/Abstract] OR "stomach cancer"[Title/Abstract] OR "gastrointestinal cancer"[Title/Abstract] OR "digestive system neoplasms"[Title/Abstract] OR "gastrointestinal tumors"[Title/Abstract]) AND ("epithelial differentiation"[Title/Abstract] OR "lineage marker"[Title/Abstract] OR "tumor phenotype"[Title/Abstract] OR "immunohistochemistry"[Title/Abstract])	English language; Publication date from January 2000 to December 2024
**Web of Science Core Collection**	TS=("VSIG1" OR "V-set and immunoglobulin domain containing 1") AND TS=("gastric cancer" OR "stomach cancer" OR "gastrointestinal cancer" OR "digestive system neoplasms" OR "gastrointestinal tumors") AND TS=("epithelial differentiation" OR "lineage marker" OR "tumor phenotype" OR "immunohistochemistry")	Document types: Article OR Review; English language; Publication years 2000–2024
**Scopus**	(TITLE-ABS-KEY("VSIG1" OR "V-set and immunoglobulin domain containing 1")) AND (TITLE-ABS-KEY("gastric cancer" OR "stomach cancer" OR "gastrointestinal cancer" OR "digestive system neoplasms" OR "gastrointestinal tumors")) AND (TITLE-ABS-KEY("epithelial differentiation" OR "lineage marker" OR "tumor phenotype" OR "immunohistochemistry"))	English language; Publication years 2000–2024

Supplementary Table S1. Database-Specific Search Strategies. Searches were conducted between January 2000 and December 2024. No initial restrictions regarding study design were applied. Reference lists of included articles were manually screened to identify additional relevant publications not captured by database queries.
